# Hydatid disease of proximal femur treated using a mega prosthesis: A case report

**DOI:** 10.1016/j.ijscr.2020.02.002

**Published:** 2020-02-06

**Authors:** Achraf Oueslati, Khalil Amri, Mohamed Ali Chefi, Youssef Mallat, Tallel Znagui, Lotfi Nouisri

**Affiliations:** Department of Orthopaedic Surgery and Traumatology, Military Hospital of Instruction of Tunis, Tunisia

**Keywords:** Bone hydatidosis, Femur, Mega prosthesis

## Abstract

**Introduction:**

Bone hydatidosis is a rare disease. It is characterised by discreet and nonspecific symptomatology that makes the diagnosis late and the treatment difficult to manage.

**Presentation of case:**

We report the case of a 29-year-old woman hospitalised for right hip pain with a lytic image well limited in the upper extremity of the right femur. We retained the diagnosis of a unicameral bone cyst which weakens the cortical bone. Osteosynthesis with a Gamma nailing was indicated to prevent fractures. At five years of follow up, the patient presented a pain recurrence. Radiographs showed almost a destruction of the upper extremity of the femur and a loosening of osteosynthesis material. Thus, we indicated the removal of the osteosynthesis material and a bone biopsy were performed. Histopathological examination leads to an osseous hydatid cyst diagnosis. We decide to carry out a carcinological resection and reconstruction with a mega prosthesis. The clinical results were excellent and no recurrence for three years follow-up.

**Discussion:**

Bone hydatidosis is challenging to diagnose at an early stage, mainly when it simulates a bone cyst. Moreover, the consequence of a late or misdiagnosed bone hydatidosis is devastating. Because of its diffuse and infiltrative character, it gradually invades the whole bone, which makes it more complicated to manage.

**Conclusion:**

Bone hydatidosis is a rare disease that can simulate other pathologies like a bone cyst in our case. In these situations, the anamnesis has a primordial place to guide the diagnosis and investigations.

## Highlights

• Bone Hydatidosis has nonspecific clinical and radiological signs.
• These characters may lead to late or misdiagnosis situation.
• Anamnesis is very important, it could guide the diagnosis.
• The diffuse and infiltrative characters of bone hydatidosis make it challenging to manage.
• The reconstruction prosthesis allows extensive resection without damaging the function.

## 1 Introduction

Osseous hydatid disease is an involvement of the bone by an anthropozoonosis, caused by the larval form of the tapeworm Echinococcus granulosus [1]. Even in endemic areas, bone hydatidosis contributing only 0.5–2.5% of all hydatid cysts [2,3].

It is challenging to manage because the symptoms are generally late, and the radiological images are not specific. As a result, the diagnosis is often made only at the late stage of extension of the parasitic osteitis, which makes the treatment difficult [4], and the prognosis is so severe that this condition has been called “white cancer” [5].

We report here a case of a misdiagnosed hydatidosis of the upper extremity of the femur. Evolution was severe with a rapid widespread bony disruption. Treatment consisted of extensive resection and reconstruction by a mega prosthesis.

This work has been reported in line with the SCARE 2018 criteria [6]

## 2 Case report

**Fig. 1:**
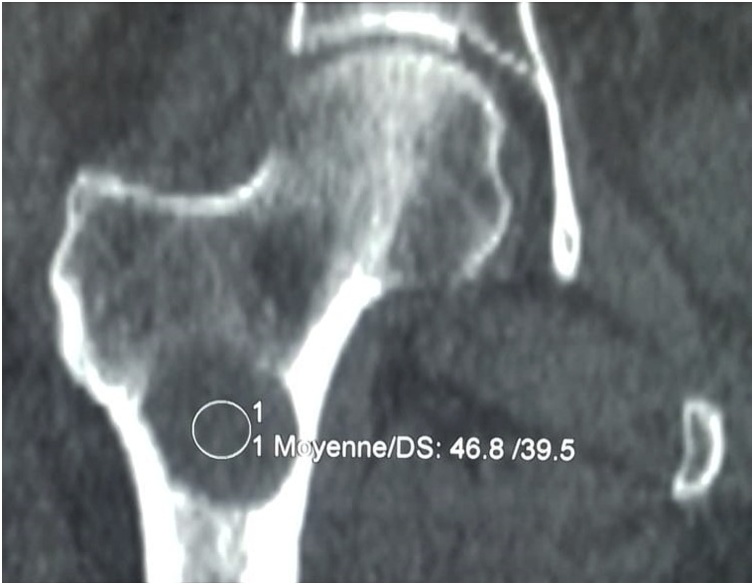
CT image of a lytic bone lesion of the upper femoral metaphysis.

We report a case of A 29-year-old woman living in a rural area and working as a farmer, with no significant pathological history. She was presented to our hospital for right hip pain. A physical examination revealed a right hip pain at mobilisation, with a normal range of motion. Plain X-rays and a Computed tomography of the right hip showed a lytic bone lesion well limited at the level of the upper metaphysis of the right femur without periosteal reaction or a weakening of the cortex (Fig. 1).

The diagnosis of the unicameral bone cyst was retained based on the radiological aspect.

A gamma nail osteosynthesis was performed to prevent the risk of fractures. The surgery had an immediate simple operative sequence, but the patient didn't present to the late follow-up.

**Fig. 2:**
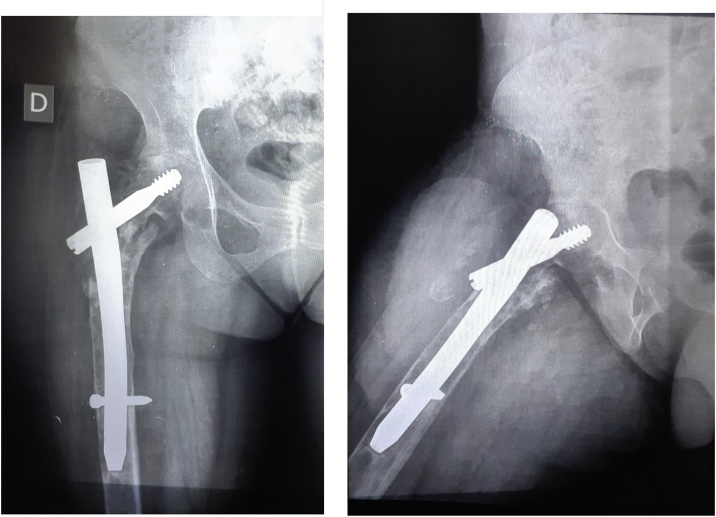
X-ray images showing the extensive lysis of the right trochanteric region and the femoral neck..

Five years later, she was admitted to our hospital for a recurrence of the right hip pain and tumefaction, which is progressively getting worse and restricting the daily activity. The physical examination revealed a tumefaction at the level of the femoral greater trochanter. The right hip joint was painful and stiff. The scar was good, and there were no signs of inflammation of the superficial skin. Conventional radiology showed extensive lysis of the right trochanteric region and the femoral neck. And the loosening of osteosynthesis material (Fig. 2).

**Fig. 3:**
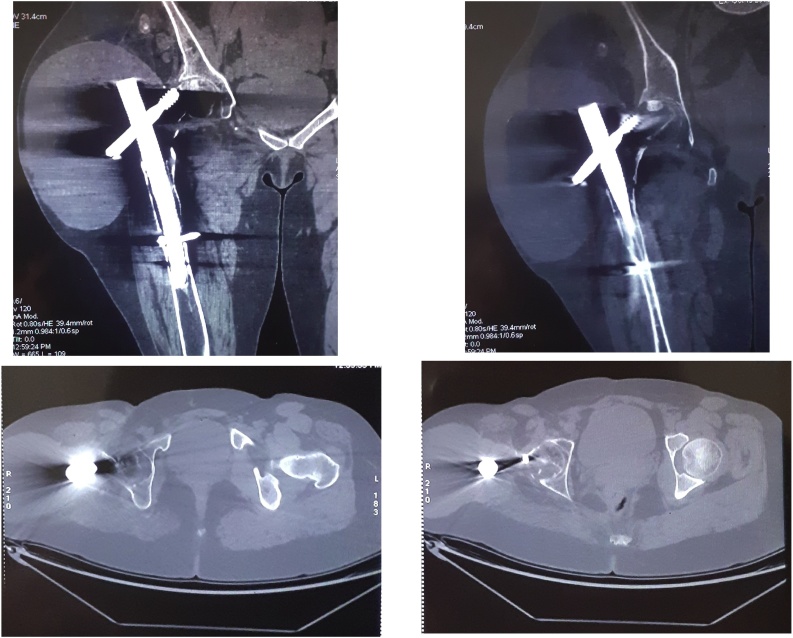
CT images showing a bone loss in the trochanteric region, and cystic lesion of the peri-trochanteric soft tissue..

Computed tomography (CT) revealed a bone loss in the trochanteric region, cystic lesion of the peri-trochanteric soft tissue, calcified in some places and with an outline delineating it from the surrounding muscles (Fig. 3). Findings were compatible with a hydatid cyst.

**Fig. 4:**
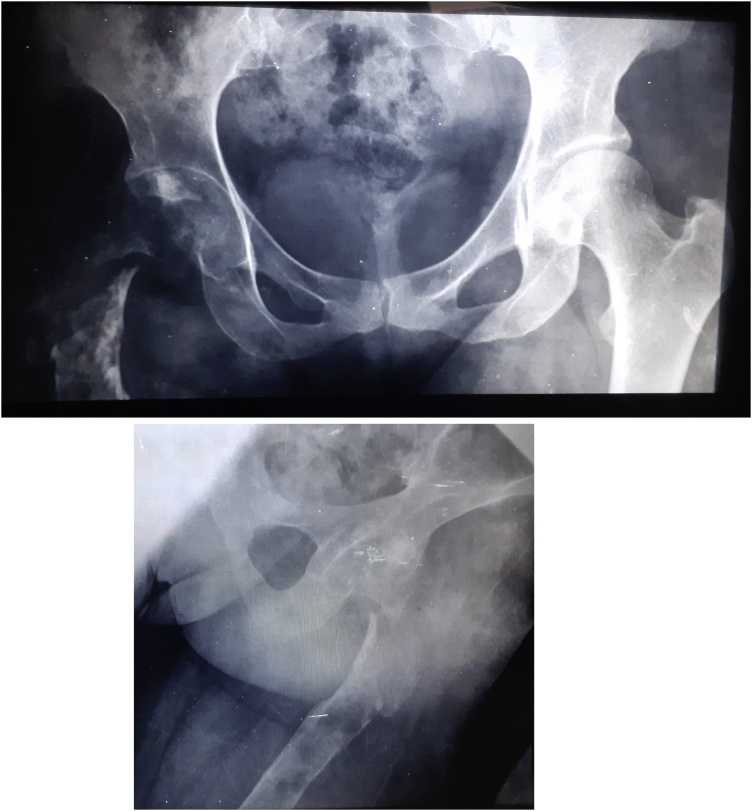
X-ray images after removing the osteosynthesis material.

So, we decided to remove the osteosynthesis material (Fig. 4) and make a bone biopsy.

The biopsy brought membranous elements of white-pearly colour, and microscopic histopathological examinations confirmed the diagnosis of a hydatid cyst.

**Fig. 5:**
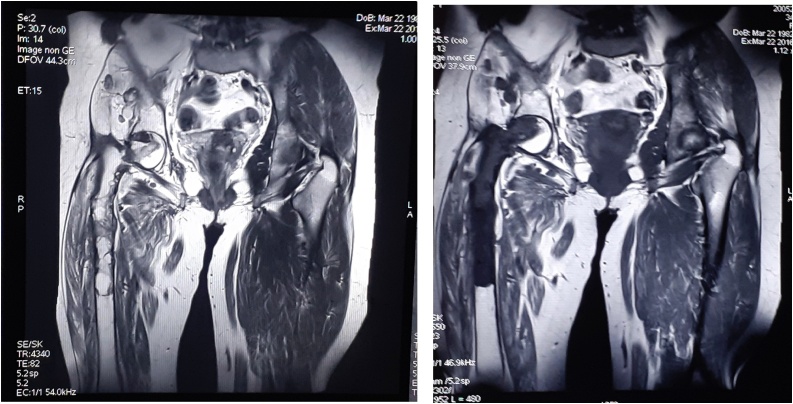
MRI images to evaluate the local extension of cystic lesions.

Magnetic resonance imaging (MRI) was performed to evaluate the local extension. It showed multiple cystic lesions. These cystic lesions were extending from the upper 1/3 of the femoral diaphysis to the epiphysis, and it doesn’t cross the joint space. In the soft parts, the lesions spread to the minimus and medius gluteus muscles (Fig. 5).

Careful clinical examination, chest X-rays and an abdominal ultrasound scan revealed no other lesions.

**Fig. 6:**
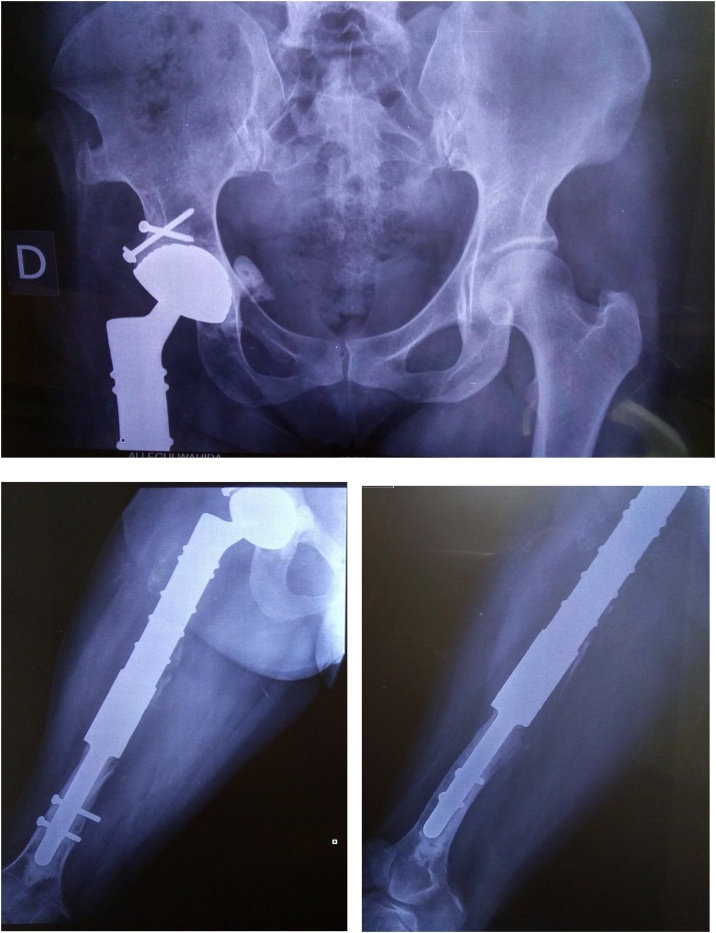
Postoperative X-ray images.

Under general anaesthesia, a senior surgeon carefully isolated the entire cyst from the surrounding muscles and excised it with 12 cm of the proximal femur. The excision was made, leaving a wide chirurgical margin, and a reconstruction by a mega prosthesis of the right hip was performed (Fig. 6).

**Fig. 7:**
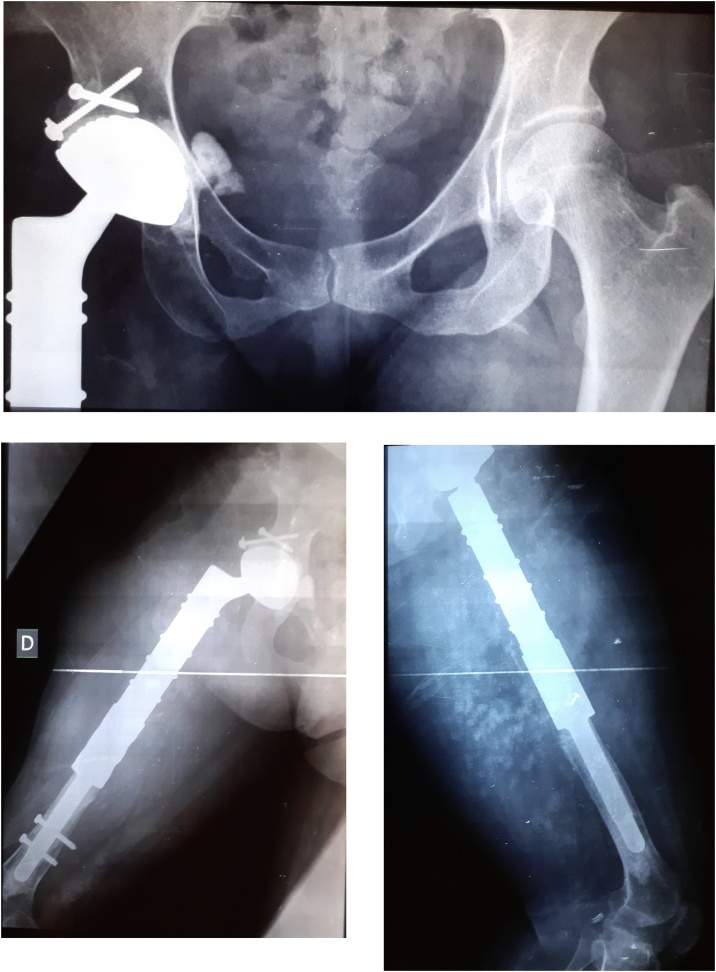
Three years postoperative X-ray images.

Postoperative recovery was remarkably uneventful, and she was able to walk with crutches. Three months later, she was walking without help. Her only problem was a 2 cm limb shortening well correct by an orthopaedic sole. she was satisfied and resumed normal daily activity without discomfort. And, there had been no recurrence three years after the cyst was removed (Fig. 7).

## 3 Discussion

Hydatid disease is a parasitic disease caused by the development in humans of the larval form of a tapeworm called Echinococcus Granulosus. Contamination occurs through the accidental ingestion of Echinococcus Granulosus eggs from contaminated food.

Its preferential localisation in the lung and the liver, Bone hydatidosis is rare; it accounts for only 0.5–2.5% of all locations [2,3].

Bone localisation has a slow and progressive evolution. But it has a diffuse and infiltrative character. It gradually invades the whole bone and adjacent soft parts, which makes it often late diagnosed and more complicated to manage.

The clinical signs are not specific, dominated by pain, swelling, and fractures, which is a significant mode of revelation.

Conventional radiology shows a cystic or irregular destruction of bone with a “honeycomb” appearance without periosteal reaction. These characteristics often cause misdiagnoses such as bone cyst and giant cell tumour of bone [7,8].

CT and MRI findings show the location, size, and appearance of the cyst. However, MRI is more reliable than CT because it shows not only the peripheral rim of the cyst and its multilocular character, but it also has a particular value in revealing the relationship of the lesion to the surrounding soft tissue [9].

In some situation, especially at an early stage, Clinical and radiological signs could be non-specific, which can lead to misdiagnosis. Like in our case, it was diagnosed as a bone cyst. This situation has serious consequences.

However, we could avoid this situation if we focus on some features like the rural origin and professional exposition of the patient, and pushing further the anamnesis. At least we would have thought about the bone hydatid cyst and investigate further. We could practice immunodiagnostics tests such as Enzyme-linked immunosorbent assay (ELISA), indirect immunofluorescence test or other tests. These tests are easy to perform and have a great sensibility and specificity [10].

The healing treatment of hydatid osteopathy requires surgery. Surgery could be conservative with curettage and aspiration of the bone cyst. But, the ideal surgery, when it is possible would be radical and perform an extensive oncological resection [11] of the cyst and surrounding bone and replacement of bone defects with bone grafts or a prosthesis. This carcinological resection avoids secondary infection, and prevent local recurrence that occurs in conservative treatment and which rate of 50% in some cases [8,12].

In our case, the massive reconstruction prosthesis allowed us to be wide at resection, without compromising the functional prognosis and quality of life of our patient.

Regarding chemotherapy, mainly albendazole, its effectiveness remains controversial, and there is no consensus on the dose and duration of treatment [13,14]. It was not adopted in our case.

## 4 Conclusion

Bone hydatidosis is a rare disease. Radiological aspect can simulate other pathologies like a bone cyst in our case, which leave to a misdiagnosis and a severe prognosis. In these situations, the anamnesis has a primordial place because some information, such as the patient's living area or his professional exposure, can guide the diagnosis and investigations.

## Declaration of Competing Interest

There are no conflicts of interest.

## Funding

There are no sources of funding for our research.

## Ethical approval

We declare that ethical approval has been given.

## Consent

A written informed consent was obtained from the patient.

## Author contribution

Achraf Oueslati: Original draft writing.

Khalil Amri: Data analysis.

Mohamed Ali Chefi: Data collection.

Youssef Mallat: Paper editing.

Tallel Znagui: Supervision.

Lotfi Nouisrif: Paper validation.

## Guarantor

Oueslati Achraf.

## Provenance and peer review

Not commissioned, externally peer-reviewed.
